# Reconfigurable spintronic logic gate utilizing precessional magnetization switching

**DOI:** 10.1038/s41598-024-65634-9

**Published:** 2024-06-26

**Authors:** Ting Liu, Xiaoguang Li, Hongyu An, Shi Chen, Yuelei Zhao, Sheng Yang, Xiaohong Xu, Cangtao Zhou, Hua Zhang, Yan Zhou

**Affiliations:** 1 College of Engineering Physics, and Shenzhen Key Laboratory of Ultraintense Laser and Advanced Material Technology, Shenzhen Technology University, Shenzhen, 518118 China; 2 College of New Materials and New Energies, Shenzhen Technology University, Shenzhen, 518118 China; 3 School of Science and Engineering, The Chinese University of Hong Kong, Shenzhen, 518172 China; 4 Research Institute of Materials Science of Shanxi Normal University & Collaborative Innovation Center for Shanxi Advanced Permanent Magnetic Materials and Technology, Linfen, 041004 China; 5 School of Chemistry and Materials Science of Shanxi Normal University & Key Laboratory of Magnetic Molecules and Magnetic Information Materials of Ministry of Education, Linfen, 041004 China

**Keywords:** Spintronics, Ferromagnetism, Electronic and spintronic devices

## Abstract

In traditional von Neumann computing architecture, the efficiency of the system is often hindered by the data transmission bottleneck between the processor and memory. A prevalent approach to mitigate this limitation is the use of non-volatile memory for in-memory computing, with spin–orbit torque (SOT) magnetic random-access memory (MRAM) being a leading area of research. In this study, we numerically demonstrate that a precise combination of damping-like and field-like spin–orbit torques can facilitate precessional magnetization switching. This mechanism enables the binary memristivity of magnetic tunnel junctions (MTJs) through the modulation of the amplitude and width of input current pulses. Building on this foundation, we have developed a scheme for a reconfigurable spintronic logic gate capable of directly implementing Boolean functions such as AND, OR, and XOR. This work is anticipated to leverage the sub-nanosecond dynamics of SOT-MRAM cells, potentially catalyzing further experimental developments in spintronic devices for in-memory computing.

## Introduction

With the rapid development of data-driven applications, the energy consumption and delay caused by data communications between memory and processor have become the major challenge for the computing system with von Neumann architecture. The concept of In-memory computing (IMC)^1–5^ has been proposed to address this bottleneck by employing the same non-volatile memory cell for both data processing and storage. Among all the solutions, spintronic devices manipulate data by controlling the spin of electrons, showing advantages in access speed, energy consumption and durability. Representative examples of such devices include spin-transfer torque (STT)^6–9^ magnetic memory and SOT^10–16^ magnetic memory. Despite the benefits in reading performance and power consumption, the SOT memory cell requires the assistance of a small in-plane external magnetic field to break the switching symmetry, which negatively impacts the integration density and stability of devices. On the other hand, although the logic gate functionality AND and OR have been proposed by adjusting the threshold switching current density of the MTJ, a straightforward solution for XOR function is still absent. Particularly, the realization of XOR function requires a pre-processing of the input signal, or otherwise the cascading of multiple memory cells, leading to a substantial increase in power consumption and the circuitry complexity^17–22^. In general, the roadmap in developing the SOT-MRAM cell for the IMC application is largely elusive up to now.

Here we propose a simple but effective scheme based on the well-understood precessional switching^23–26^ of the MTJ to realize the reconfigurable Boolean operations. Through the controlling of either the amplitude or the width of the current pulses, the 180°, 360°, and 540° magnetization switching of the free layer can be selected in a deterministic manner without the assistance of external field. This typical process of the precessional magnetization switching is further utilized to implement AND, OR, and XOR operations with a single MTJ, at a time scale of sub-nanosecond. The simulation results combining macro spin modeling and micromagnetic modeling demonstrate the predominant role of field-like torque^27–32^ in realizing the above-mentioned operations. The aim of this work is to exploit the magnetization dynamics driven by spin–orbit torque within nano-second, which has been long-neglected since the switching at this time scale is usually considered nondeterministic, and potentially catalyzing further experiments in developing spintronic devices for in-memory computation.

## Results

**Figure 1:**
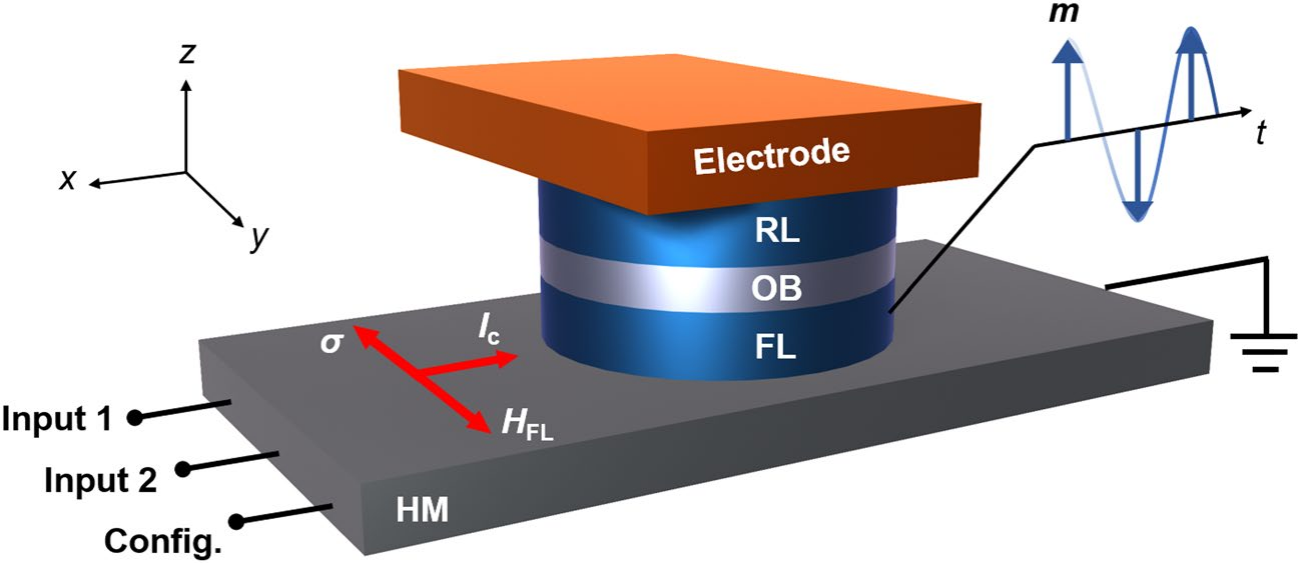
Schematic of the simulated SOT based IMC cell. Current flows into the HM along the *x* direction, and the total current density sums the Input1, Input2 and Configuration terminals. The red arrows indicate the directions of the charge current *I*_c_, the spin polarization ***σ***, and the effective field of the interfacial Rashba-Edelstein effect ***H***_FL_. The spin orbit torques drive the free layer magnetization into precession, enabling the multi-cycle switching.

Figure 1 illustrates the schematic of the proposed IMC unit, which is developed from a general SOT-MRAM cell consisting of the heavy metal layer (HM), the free layer (FL), the oxide barrier (OB), the reference layer (RL), and the top electrode. Both the FL and RL have perpendicular magnetic anisotropy (PMA). We assume the diameter of the MTJ is approximately the domain wall width, in this case the dynamics of the free layer is quasi-uniform, and the macro spin modeling is valid.

When the charge current flows through the HM in the -*x* direction, the spin of the electrons will be polarized in the -*y* direction due to the spin–orbit coupling, and exert spin torques on the adjacent free layer. The spin torques can be attributed to both the bulk Spin Hall effect, giving rise to the damping-like component, and the interfacial Rashba-Edelstein effect, giving rise to the field-like component. The magnetization dynamics of the free layer can be described by the Landau-Lifshitz-Gilbert (LLG) Eq.^33^ including the spin–orbit torques as:

1 $$\frac{{\partial {\mathbf{m}}}}{\partial t} = - \gamma {\varvec{m}} \times \left( {{\varvec{H}}_{{{\mathbf{anis}}}} + {\varvec{H}}_{{{\mathbf{thm}}}} } \right) + \alpha {\varvec{m}} \times \frac{{\partial {\varvec{m}}}}{\partial t} + \frac{{\gamma \hbar J_{{\text{c}}} }}{{2eM_{{\text{s}}} \mu_{0} t_{{\text{F}}} }}\left[ {\xi_{{{\text{DL}}}} {\varvec{m}} \times \left( {{\varvec{\sigma}} \times {\varvec{m}}} \right) + \xi_{{{\text{FL}}}} {\varvec{\sigma}} \times {\varvec{m}}} \right]$$

where $\gamma$ is the gyromagnetic ratio, $\alpha$ the Gilbert damping constant, $\hbar$ the reduced Planck constant, *J*_c_ the charge current density injected into the HM, *e* the electron charge, $\mu_{0}$ the permeability of vacuum, *M*_s_ the saturation magnetization, $t_{{\text{F}}}$ the thickness of the free layer, $\xi_{{{\text{DL}}}}$ and $\xi_{{{\text{FL}}}}$ are the efficiency constants damping-like torque and field-like torque, ${\varvec{m}}$ the magnetization unit vector of the free layer, ${\varvec{\sigma}}$ is the spin polarization vector. The origin of the damping-like SOT is the spin Hall effect, and the origin of the field-like spin torque is commonly considered a combination of the interfacial Rashba–Edelstein effect and the oersted field from the charge current^34^. We note that by the definition in Eq. (1), the efficiency constant of damping-like SOT, $\xi_{{{\text{DL}}}}$, and the efficiency constant of field-like SOT, $\xi_{{{\text{FL}}}}$, should have opposite sign to trigger the precessional switching dynamics. In this case, the two SOT components compete to align the magnetization in opposite directions. Previous experiments demonstrated the ratio $\xi_{{{\text{FL}}}} /\xi_{{{\text{DL}}}}$ = -0.7 to -1 in the Co/Pt/Al_2_O_3_ heterostructure ^35,36^. While the Ta/CoFeB/MgO heterostructure can provide a larger ratio up to $\xi_{{{\text{FL}}}} /\xi_{{{\text{DL}}}}$ = -4 ^26,37,38^. We assume the HM is Ta, the FL is CoFeB, and the corresponding parameters we used for simulation are listed in Table 1.

**Table 1:** Simulation parameters used for the macro spin modeling.

Parameter	Symbol	Value
Free layer thickness	*t*_F_	1 nm
Effective PMA constant	${K}_{\text{eff}}$	7 $\times$ 10^4^ J/m^3^
Damping constant	*α*	0.1
Saturation magnetization	*M*_s_	2.0 $\times$ 10^6^ A/m
Temperature	*T*	300 K
Damping-like SOT efficiency	${\xi }_{\text{DL}}$	− 0.1
Field-like SOT efficiency	${\xi }_{\text{FL}}$	0.1–0.2

**Figure 2:**
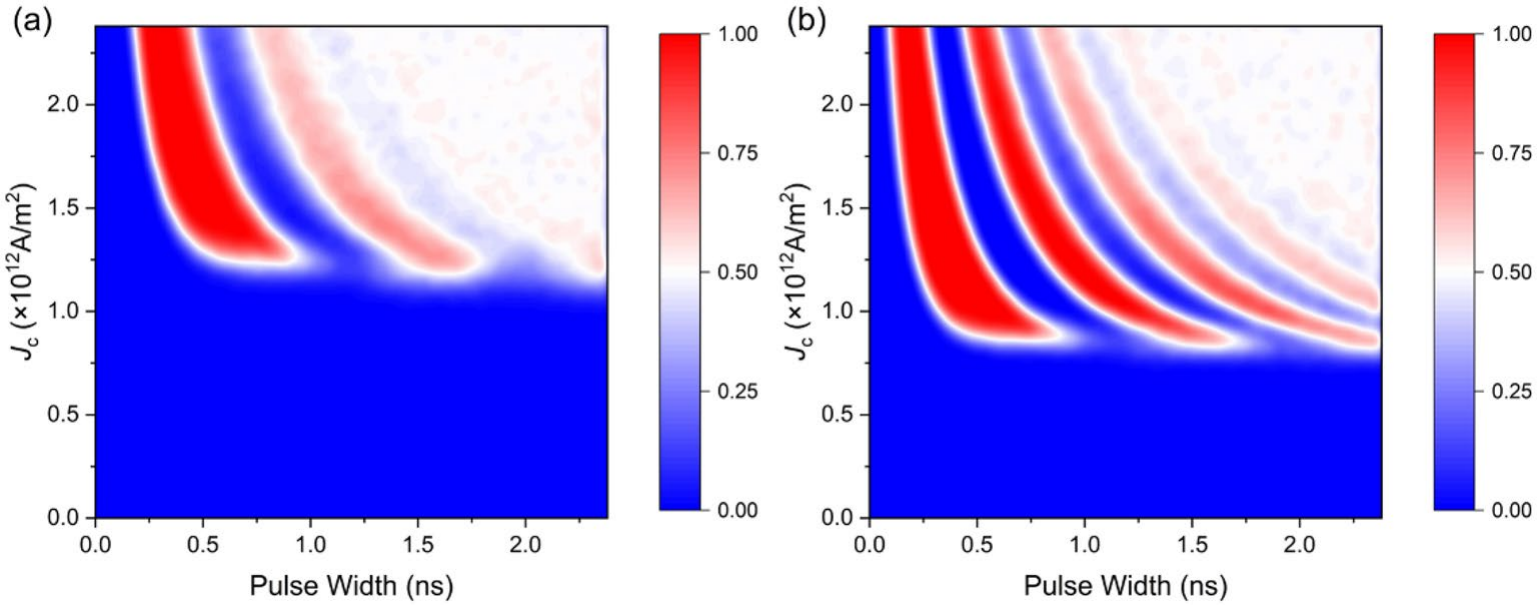
Magnetization switching probability as a function of the current density and pulse width. (**a**) ${\xi }_{\text{FL}}$ = 0.1 and (**b**) ${\xi }_{\text{FL}}$ = 0.2. The magnetization is fully relaxed to reach ± *z* direction when the current is turned off. And the switching probability *p*_sw_ is obtained by counting the switching times from 200 trials with the parameters listed in Table 1.

We first used a single shot current pulse with varied current density *J*_c_ and pulse width *T*_c_ to excite the magnetic dynamics of the free layer at room temperature, and calculated the magnetization switching probability *p*_sw_ averaged over 200 trails. For the case where the damping-like and the field-like components of the SOT having opposite sign and equal strength ($\xi_{{{\text{DL}}}} = - 0.1$ and $\xi_{{{\text{FL}}}} = 0.1$), the deterministic switching (*p*_sw_ > 0.95) only occurs when *T*_c_ < 1 ns, as shown in Fig. 2a. The increasing of *T*_c_ will first prohibit the magnetization switching, and create a gap where *p*_sw_ = 0, and then set stochastic switching (*p*_sw_ = 0.5). The switching probability for the case $\xi_{{{\text{DL}}}} = - 0.1,\xi_{{{\text{FL}}}} = 0.2$ is shown in Fig. 2b. The increased field-like SOT effectively reduces the threshold current density from 1.2 × 10^12^ A/m^2^ to about 0.8 × 10^12^ A/m^2^. More importantly, we observed a second branch in the current parameter space where the switching is deterministic. The result indicates the dominance of the field-like SOT will lead to magnetization precession around the direction of the spin polarization, agrees with previous experimental findings^23,26^. We also note that this phenomenon is a representation of binary memristivity, since the resistance of the cell alternates between two states as the current density, or the pulse width increasing.

**Figure 3:**
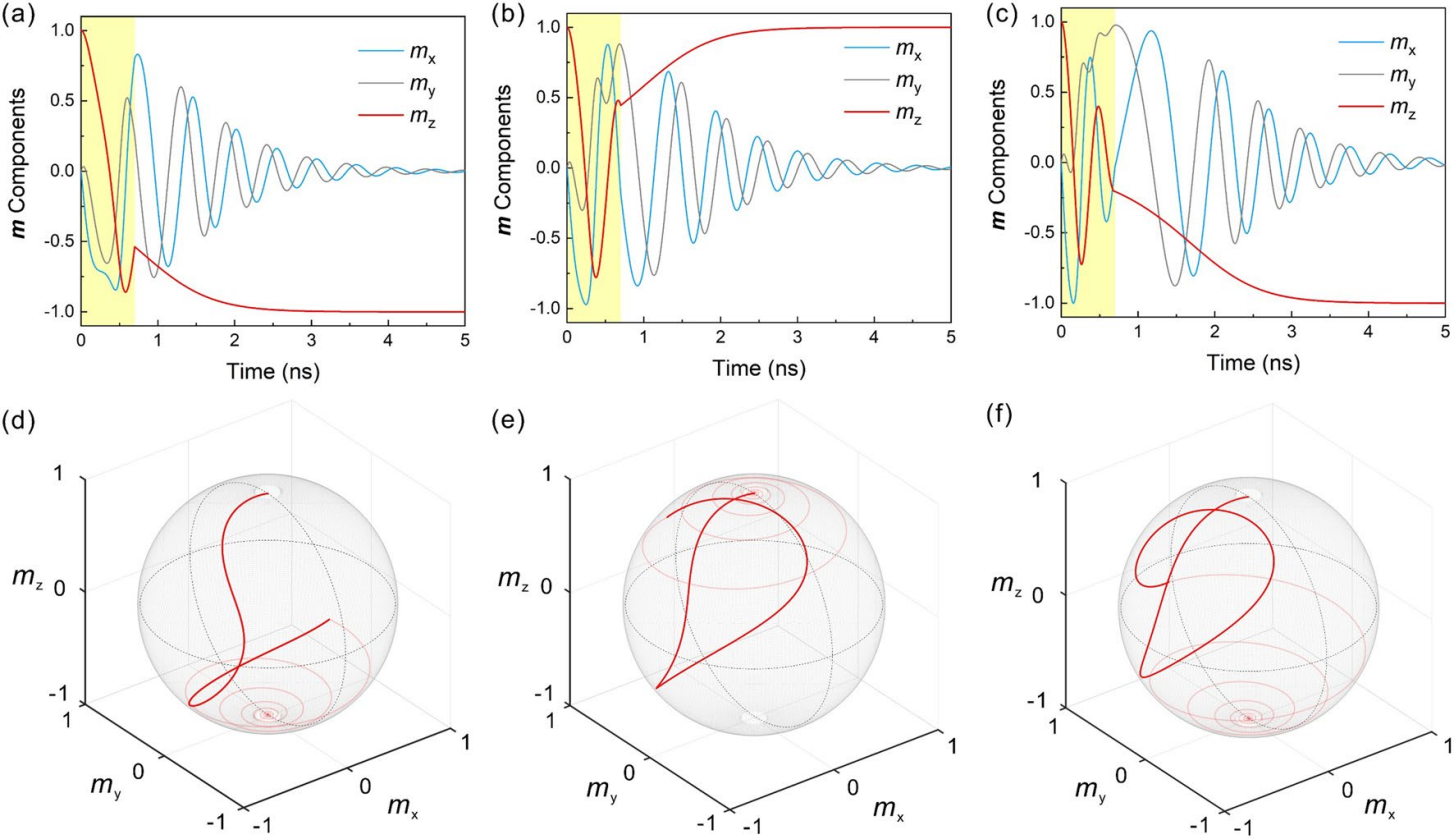
Precessional magnetization switching processes driven by the combined damping-like and field-like SOT. (**a**)–(**c**) Evolution of the magnetization components with time. The duration of the current pulse is highlighted by the yellow part. The current pulse width *T*_c_ = 0.7 ns, and the current density *J*_c_ = (**a**) 0.94 $\times$ 10^12^ A/m^2^, (**b**) 1.24 $\times$ 10^12^ A/m^2^ and (**c**) 1.7 $\times$ 10^12^ A/m^2^. (**d**)–(**f**) Magnetization trajectories corresponding to (**a**)–(**c**). We highlight the dynamics excited by current pulses in solid, and the relaxation dynamics in transparent.

We further fixed the pulse width *T*_c_ = 0.7 ns, and investigated the detailed magnetization switching processes at zero temperature with increasing *J*_c_. As shown in Figs. 3a and d, when *J*_c_ = 0.94 × 10^12^ A/m^2^ the SOT overcomes the perpendicular magnetic anisotropy, and drives the magnetization over the energy barrier. When the excitation stops, the magnetization precesses to the -*z*-direction and realizes the 180° switching, representing the general dynamics for the current configuration located in the first branch of deterministic switching in Fig. 2b. When the current density is increased to 1.24 × 10^12^ A/m^2^, as shown in Fig. 3b and e, the field-like component of SOT drives the magnetization into in-plane precession, and completes the 360° switching. In this case, the magnetization direction of the free layer does not change. When the current amplitude is further increased to 1.7 × 10^12^ A/m^2^, as shown in Fig. 3c and f, the in-plane precession completes the 540° switching, representing the dynamics for current configuration in the second branch of deterministic switching in Fig. 2b. Besides, we have verified the switching stability by including 5 ns thermal fluctuation before the charge current is applied (see Supplementary Material Fig. S1). Here we denote the critical current density that enables the magnetization switching of 180°, 360°, 540° as *J*_π_, *J*_2π_, and *J*_3π_, respectively.

**Figure 4:**
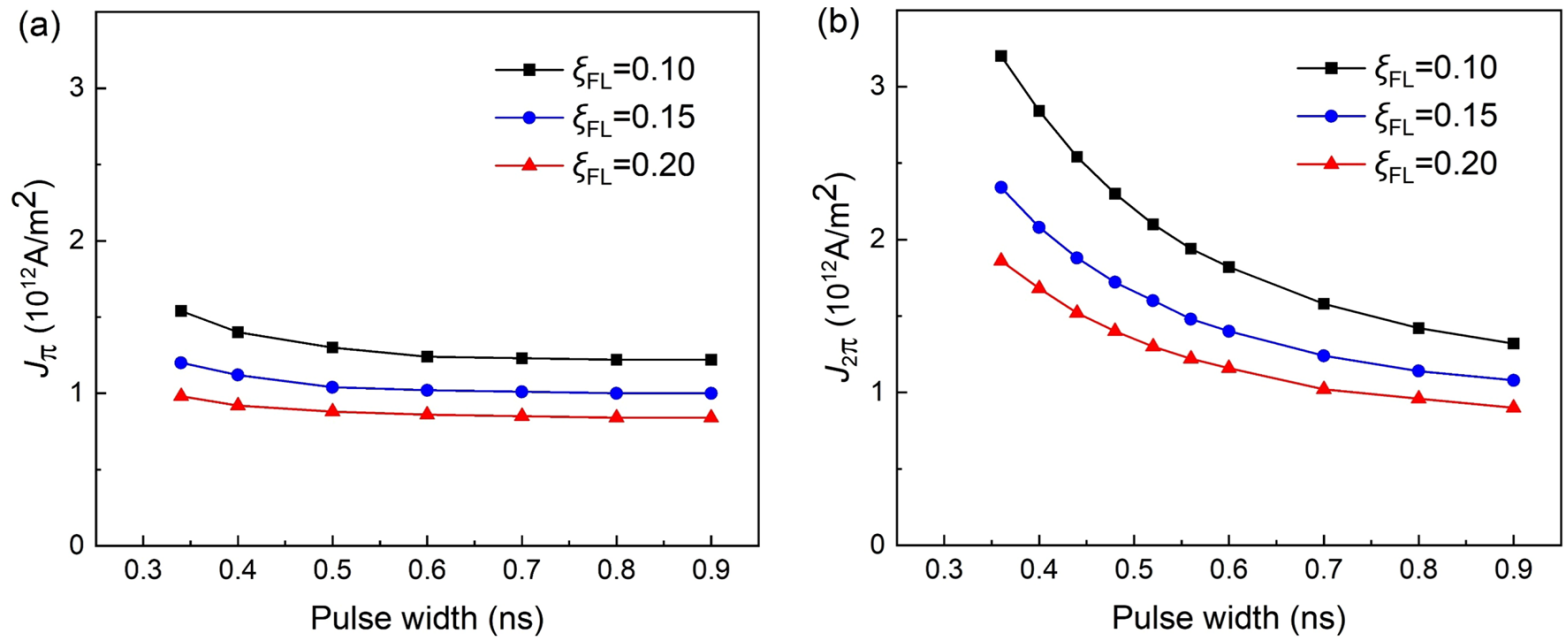
Critical current density vs. pulse width with varied field-like torque. The relationship between the critical current density of (**a**) 180° and (**b**) 360° switching with the pulse width *T*_c_.

Both the field-like SOT and the current pulse width have pronounced impacts on the precessional magnetization switching dynamics. We further investigated the critical current density as a function of the pulse width with varying $\xi_{{{\text{FL}}}}$, as shown in Fig. 4. For both the cases of 180° switching and 360° switching, the increasing $\xi_{{{\text{FL}}}}$ effectively reduces the critical current densities. On the other hand, the dependences of *J*_π_ and *J*_2π_ on *T*_c_ are quite different. As shown in Fig. 4a, *J*_π_ changes sightly when *T*_c_ > 400 ps. However, *J*_2π_ nonlinearly decreases with the pulse width, and approaches *J*_π_ when *T*_c_ > 1 ns, as shown in Fig. 4b. The results indicate that the precessional switching is actually a dynamics process, and can only be excited by short current pulses, since the current window for deterministic switching quickly closes and leads to stochastic switching as the pulse width increases.

We used the open-source micromagnetic simulator Mumax3^39^ to confirm the precessional magnetization switching dynamics demonstrated by the macro spin modeling. The parameters we adopted for the micromagnetic simulation are the same to those listed in Table 1. We assume the diameter of the FL is 50 nm, and the mesh size is 1 nm × 1 nm × 1 nm. The exchange constant *A*_ex_ = 1 × 10^–11^ J/m, the PMA energy density *K*_u_ = 2.36 × 10^6^ J/m^3^, and the corresponding effective PMA energy density *K*_eff_ = 7.3 × 10^4^ J/m^3^. The strength of damping-like and field-like spin–orbit torques are adjusted by tuning the secondary Slonczewski term. In particular, we use Slonczewski spin transfer torque to replace spin orbit torque in Mumax3 by setting ${\Lambda }$ = 1, *P* = $\xi_{{{\text{DL}}}}$, $\epsilon {\prime } = \eta \epsilon$, *ϵ* defined as:

2 $$\epsilon = \frac{{P\left( {{\text{r}},{\text{t}}} \right){\Lambda }^{2} }}{{\left( {{\Lambda }^{2} + 1} \right) + \left( {{\Lambda }^{2} - 1} \right)\left( {{\varvec{m}} \times {\varvec{\sigma}}} \right)}}$$

**Figure 5:**
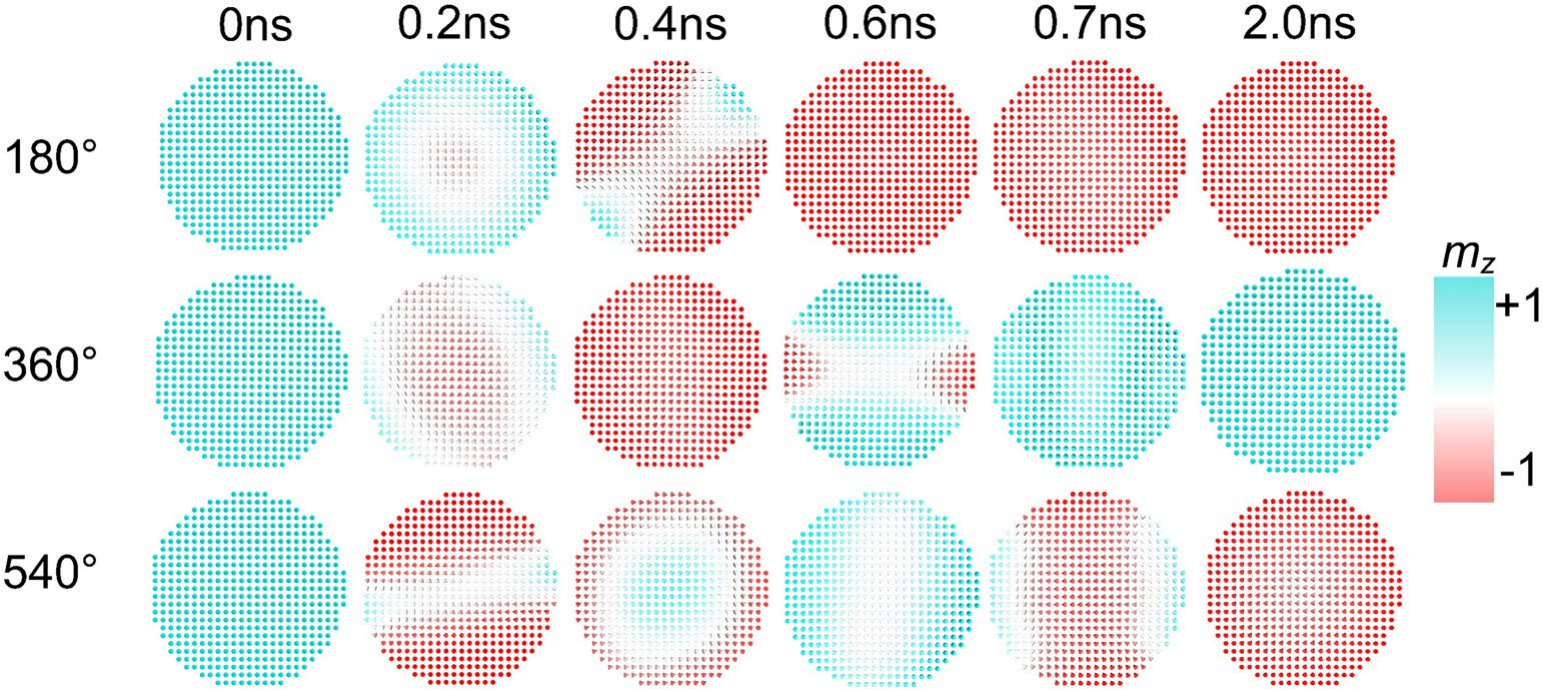
Snapshots of the magnetization distribution at different time during the switching process. From the top to the bottom, 180°, 360°, and 540° switching are excited by the current pulses with a density of 1.0 $\times$ 10^12^ A/m^2^, 1.5 $\times$ 10^12^ A/m^2^, 2.0 $\times$ 10^12^ A/m^2^, respectively.

***m*** is the FL magnetization, ${\varvec{\sigma}}$ the spin polarization vector, *P* the spin polarization, $\epsilon {\prime }$ the secondary Slonczewski STT term, ${\Lambda }$ the barrier layer thickness, $\xi_{{{\text{FL}}}} = \eta \xi_{{{\text{DL}}}}$. We excited the FL by a current pulse of 0.7 ns, and then relax the magnetization for 10 ns. In this case, the threshold current densities for 180°, 360° and 540° switching are *J*_π_ = 0.88 × 10^12^ A/m^2^, *J*_2π_ = 1.21 × 10^12^ A/m^2^, *J*_3π_ = 1.97 × 10^12^ A/m^2^, respectively. They are increased compared to the those obtained by macro spin modeling, and could be attributed to the slightly non-coherent switching of the free layer. Figure 5 shows the free layer magnetization evolution of the 180°, 360° and 540° switching, which also captures the non-coherent magnetization distribution during the switching process. We observed that the domain first nucleates in the middle and then propagates outward. This may leverage the exchange energy and the threshold current density for multicycle magnetization switching as well. However, for the MTJ with a diameter smaller than the domain wall width, the precessional switching dynamics will not be qualitatively affected by demagnetization and exchange.

Utilizing the precessional switching mechanism, a spintronic logic gate integrating reconfigurable AND, OR and XOR operations can be implemented. The logic operation to be performed can be selected by a configuring current pulse with density of *J*_config_. The two binary logic inputs are single current pulse with the current density of *J*_in1_ and *J*_in2_, and the pulse width *T*_c_. Here we assume these current pulses are additive, which can be realized by parallel connection of the inputs, and in this case the total current density in the heavy metal layer is *J*_c_ = *J*_config_ + *J*_in1_ + *J*_in2_. The logic output can be read from the resistance of the MTJ. Noting that no external field is introduced to break the switching symmetry, thus the magnetic switching of the free layer is nonpolar, and is independent of the initial magnetization (Please see Supplementary Material Fig. S2). For simplicity, we assume the output being “0” if the resistance state of the MTJ is not changed, while the output being “1” if the high resistance state changes to low resistance state, or vice versa. In previous researches, the logic operation based on a single MTJ requires a reset process to initialize the magnetization state^20,40^. This is also realizable in our proposed scheme by introducing magnetic field to break the switching symmetry. We also highlight that the implementation of the logic operation XOR directly utilizes the 360° switching, which is actually the precessional dynamics introduced by the field-like SOT. For the example hereafter, we calculated that when *T*_c_ = 0.7 ns, the zero-temperature critical current density *J*_π_ = 0.88 × 10^12^ A m^-2^, *J*_2π_ = 1.04 × 10^12^ A/m^2^, *J*_3π_ = 1.48 × 10^12^ A/m^2^. The required current settings for achieving different logic operations are listed in Table 2.

**Table 2:** Current configurations for logic gates implementation.

	*J*_config_ (10^12^ A/m^2^)	*J*_in_ (10^12^ A/m^2^)	*T*_c_ (ns)
AND gate	0	0.5	0.7
OR gate	0.5	0.5	0.7
XOR gate	0.4	0.5	0.7

**Figure 6:**
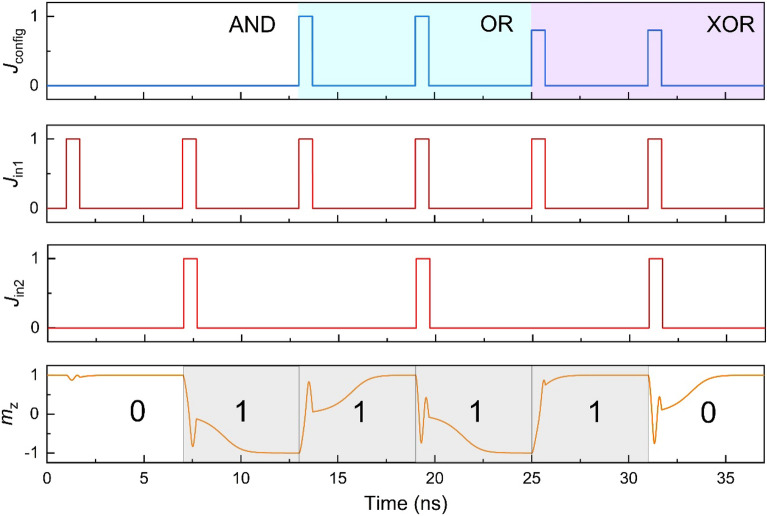
The implementation of AND, OR and XOR logic operations. The total current density in the heavy metal layer is *J*_config_ + *J*_in1_ + *J*_in2_. The bottom panel shows the magnetization response to the current pulses, and the logic outputs '0' and '1' are based on whether the magnetization switches. The current densities in this figure are normalized by 0.5 × 10^12^ A/m^2^.

As shown by the left part of Fig. 6, the logic AND function can be realized by setting *J*_config_ = 0 A/m^2^, and the operation is as follows: when *J*_in1_ = *J*_in2_ = 0 A/m^2^, corresponding to the logic input '0,0', *J*_c_ is less than the 180° switching critical current. The magnetization of the FL does not switch, and the logic output is ‘0’; When current pulse passes through either the input, i.e. *J*_in1_ = 0.5 × 10^12^ A/m^2^ or *J*_in2_ = 0.5 × 10^12^ A/m^2^, corresponding to the logic input '1,0' or '0,1'. In this case *J*_c_ = 0.5 × 10^12^ A/m^2^ < *J*_π_, the magnetization does not switch, and the logic output is ‘0’; When *J*_in1_ = *J*_in2_ = 0.5 × 10^12^ A/m^2^, corresponding to the logic input '1,1', *J*_π_ < *J*_c_ = 1.0 × 10^12^ A/m^2^ < *J*_2π_, the magnetization of the free layer deterministically switching, and the logic output is ‘1’.

The logic operation OR can be realized by setting *J*_config_ = 0.5 × 10^12^ A/m^2^, as shown in the middle part of Fig. 6. When there is no logic input current, *J*_c_ = *J*_config_ + *J*_in1_ + *J*_in2_ = 0.5 × 10^12^ A/m^2^ < *J*_π_, the logic output is ‘0’; When the current pulse passes through either the logic input, corresponding to the logic input '1,0' or '0,1', *J*_π_ < *J*_c_ = 1.0 × 10^12^ A/m^2^ < *J*_2π*,*_ the magnetization switches and the logic output is ‘1’; When *J*_in1_ = *J*_in2_ = 0.5 × 10^12^ A/m^2^, the logic input is '1,1', and *J*_c_ = 1.5 × 10^12^ A/m^2^ > *J*_3π_. In this case, the magnetization switches 540°, the resistance is changed, and the logic output is ‘1’.

Similarly, the logic operation XOR can be realized by setting *J*_config_ = 0.4 × 10^12^ A/m^2^, as shown in the right part of Fig. 6. When there is no logic input current, *J*_c_ = 0.4 × 10^12^ A/m^2^ < *J*_π_, the logic output is ‘0’; When the logic inputs are '1,0' or '0,1', *J*_π_ < *J*_c_ = *J*_config_ + *J*_in1_ + *J*_in2_ = 0.9 × 10^12^ A/m^2^ < *J*_2π_, the magnetization switches 180°, the logic output is ‘1’; When *J*_in1_ = *J*_in2_ = 0.5 × 10^12^ A/m^2^, the logic input is '1,1', *J*_2π_ < *J*_c_ = 1.4 × 10^12^ A/m^2^ < *J*_3π_, the magnetization switches 360°, in this case the resistance does not change, and the logic output is ‘0’.

We further denote the configuration current densities for realizing logic gate AND, OR and XOR by *J*_and_, *J*_or_, and *J*_xor_, respectively. For the implementation of the AND gate, *J*_and_ = 0 A/m^2^, and the current density should satisfy the following relationship:

3 $$\left\{ {\begin{array}{*{20}l} {0 \le J_{{{\text{and}}}} < J_{{\uppi }} } \hfill \\ {0 \le J_{{{\text{and}}}} + J_{{{\text{in}}}} < J_{{\uppi }} } \hfill \\ {J_{{\uppi }} \le J_{{{\text{and}}}} + 2J_{{{\text{in}}}} < J_{2\pi } } \hfill \\ \end{array} } \right.$$

For the implementation of OR gate:

4 $$\left\{ {\begin{array}{*{20}l} {0 \le J_{{{\text{or}}}} < J_{{\uppi }} } \hfill \\ {J_{{\uppi }} \le J_{{{\text{or}}}} + J_{{{\text{in}}}} < J_{2\pi } } \hfill \\ {J_{{3{\uppi }}} \le J_{{{\text{or}}}} + 2J_{{{\text{in}}}} } \hfill \\ \end{array} } \right.$$

For the implementation of XOR gate:

5 $$\left\{ {\begin{array}{*{20}l} {0 \le J_{{{\text{xor}}}} < J_{{\uppi }} } \hfill \\ {J_{{\uppi }} \le J_{{{\text{xor}}}} + J_{{{\text{in}}}} < J_{2\pi } } \hfill \\ {J_{{2{\uppi }}} \le J_{{{\text{xor}}}} + 2J_{{{\text{in}}}} < J_{3\pi } } \hfill \\ \end{array} } \right.$$

Solving the above inequalities, we have 0.49 × 10^12^ A/m^2^ ≤ *J*_in_ ≤ 0.50 × 10^12^ A/m^2^, 0.50 × 10^12^ A/m^2^ ≤ *J*_or_ < 0.54 × 10^12^ A/m^2^, 0.39 × 10^12^ A/m^2^ ≤ *J*_xor_ < 0.48 × 10^12^ A/m^2^. We performed further investigation and find that by leveraging *J*_and_, utilizing 360° and 540° switching to realize the AND gate functionality, the critical current variability can be effectively improved. In this way, Eq. (3) will be replaced by:

6 $$\left\{ {\begin{array}{*{20}l} {0 \le J_{{{\text{and}}}} < J_{{\uppi }} } \hfill \\ {J_{{2{\uppi }}} \le J_{{{\text{and}}}} + J_{{{\text{in}}}} < J_{{3{\uppi }}} } \hfill \\ {J_{{3{\uppi }}} \le J_{{{\text{and}}}} + 2J_{{{\text{in}}}} } \hfill \\ \end{array} } \right.$$

**Figure 7:**
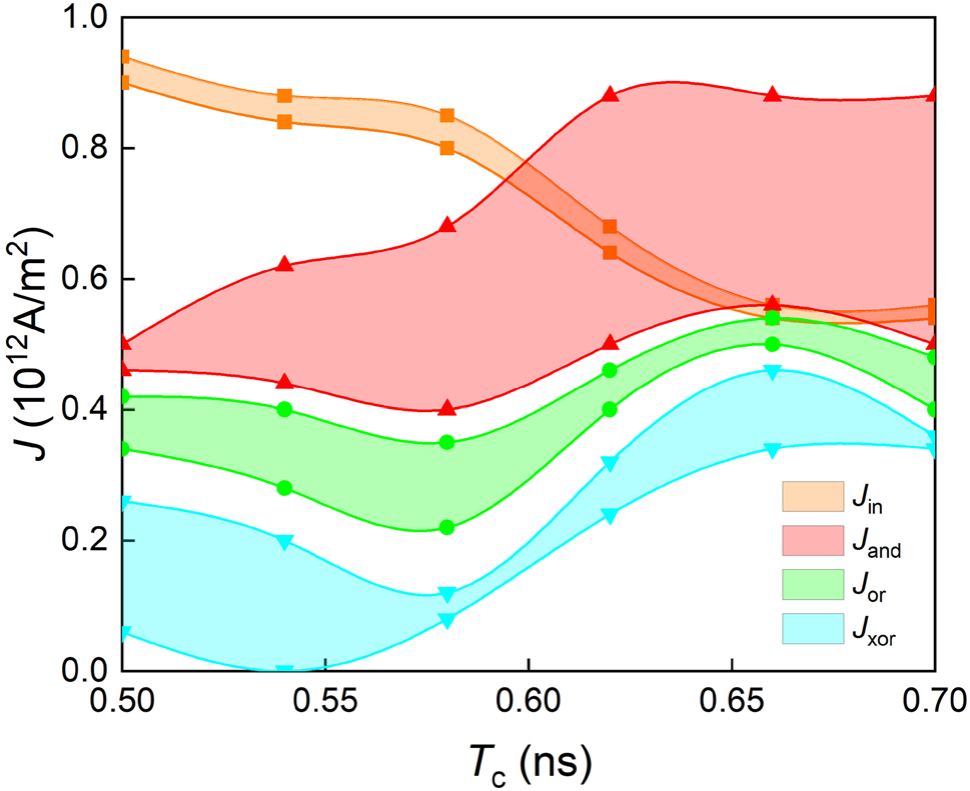
The current variability when the pulse width from 0.5 ns to 0.7 ns. The orange, red, green and blue regions represent the variability of *J*_in_, *J*_and_, *J*_or_, and *J*_xor_, respectively.

The calculated range of the current densities are sensitive to the variation of the pulse width *T*_c_, as shown in Fig. 7. The results indicate that the logic input current density *J*_in_ should be precisely controlled, and the optimized variability is about 6% when the current pulse width *T*_c_ = 0.58 ns. Correspondingly, the variability of *J*_and_, *J*_or_ and *J*_xor_ are 52%, 46% and 40%.

## Conclusions

In this study, we conducted numerical simulations to investigate the magnetization switching of the Ta/CoFeB/MgO/CoFeB heterostructure, driven by spin–orbit torque. By controlling either the amplitude or the width of the current pulses, we achieved 180°, 360°, and 540° magnetization switching without the need for an external field. A critical element in this mechanism is the field-like torque efficiency, which serves two key functions: it reduces the critical switching current density and induces an oscillatory switching behavior. Building on these findings, we propose a reconfigurable multi-functional logic gate. This gate can efficiently perform Boolean operations, including AND, OR, and XOR, without the necessity for cascading.

## Method

### Macro spin modeling

7 $$\frac{{\partial {\varvec{m}}}}{\partial t} = - \gamma \mu_{0} {\varvec{m}} \times {\varvec{H}}_{{{\mathbf{eff}}}} + \alpha {\varvec{m}} \times \frac{{\partial {\varvec{m}}}}{\partial t} + {\varvec{\tau}}_{{{\mathbf{SOT}}}}$$

8 $${\varvec{\tau}}_{{{\mathbf{SOT}}}} = \user2{ }\frac{{\gamma \hbar J_{{\text{c}}} }}{{2eM_{{\text{s}}} t_{{\text{F}}} }}\left[ {\xi_{{{\text{DL}}}} {\varvec{m}} \times \left( {{\varvec{\sigma}} \times {\varvec{m}}} \right) + \xi_{{{\text{FL}}}} {\varvec{\sigma}} \times {\varvec{m}}} \right]$$

Here, ${\varvec{\tau}}_{{{\mathbf{SOT}}}}$ is the SOT torque including the damping-like component and the field-like component, ***H***_**eff**_ the effective magnetic field, ***m*** the magnetization unit vector of the free layer, ${\varvec{\sigma}}$ the spin polarization vector, $\gamma$ the gyromagnetic ratio, $\alpha$ the Gilbert damping constant, $\hbar$ the reduced Planck constant, $J_{{\text{c}}}$ the charge current density injected into the HM, *e* the electron charge, $\mu_{0}$ the permeability of vacuum, $M_{{\text{s}}}$ the saturation magnetization, $t_{{\text{F}}}$ the thickness of the free layer, $\xi_{{{\text{DL}}}}$ and $\xi_{{{\text{FL}}}}$ are the efficiency constants damping-like torque and field-like torque.

Equation (7) is transformed to the integrable form:

9 $$\frac{{\partial {\varvec{m}}}}{\partial t} = \frac{\gamma }{{1 + \alpha^{2} }}\left[ { - {\varvec{m}} \times {\varvec{H}}_{{{\mathbf{eff}}}} - \alpha {\varvec{m}} \times \left( {{\varvec{m}} \times {\varvec{H}}_{{{\mathbf{eff}}}} } \right) + \left( {a_{{\text{J}}} + \alpha b_{{\text{J}}} } \right){\varvec{m}} \times \left( {{\varvec{m}} \times {\varvec{\sigma}}} \right) + \left( {b_{{\text{J}}} - \alpha a_{{\text{J}}} } \right){\varvec{m}} \times {\varvec{\sigma}}} \right]\user2{ }$$

with $a_{J} = \frac{{\xi_{DL} \hbar J_{{\text{c}}} }}{{2eM_{{\text{s}}} \mu_{0} t_{{\text{F}}} }}$ and $b_{J} = \frac{{\xi_{FL} \hbar J_{{\text{c}}} }}{{2eM_{{\text{s}}} \mu_{0} t_{{\text{F}}} }}$. ***H***_**eff**_ is the sum of here, ${\varvec{H}}_{{{\mathbf{anis}}}} = \frac{{2K_{eff} }}{{\mu_{0} M_{s} }}$ is the effective field of the PMA, and ${\varvec{H}}_{{{\mathbf{thm}}}} = \left( {\frac{{2\alpha k_{b} T}}{{\gamma M_{s} V_{FL} }}} \right)^{\frac{1}{2}} I_{{{\text{ran}}}}$ is a Gaussian random field ^41^ representing the influence from the temperature, here *T* is the temperature, $k_{{\text{b}}}$ is the Boltzmann constant, $V_{{{\text{FL}}}}$ is the volume of free layer, $I_{{{\text{ran}}}}$ is a random Gaussian variable with mean of 0 and standard deviation of 1. Equation (7) is numerically solved using the second order Huen’s method to include the thermal fluctuations, with a time step of 1 $\times$ 10^–13^ ns.

### Micromagnetic simulation

Our simulation is performed by the MuMax3 program. Based on the LLG equation, the magnetization under the current-induced SOT is expressed as:

Setting $a_{{\text{J}}} = \frac{{\xi_{DL} \hbar J_{{\text{c}}} }}{{2eM_{{\text{s}}} t_{{\text{F}}} }}$, $b_{{\text{J}}}$ = $\eta a_{{\text{J}}}$, $\xi_{{{\text{FL}}}} = \eta \xi_{{{\text{DL}}}}$. Equation (7) can be transformed as:

10 $$\frac{{\partial {\varvec{m}}}}{\partial t} = - \gamma \mu_{0} {\varvec{m}} \times {\varvec{H}}_{{{\mathbf{eff}}}} - a_{{\text{J}}} \gamma {\varvec{m}} \times \left( {{\varvec{m}} \times {\varvec{\sigma}}} \right) - b_{{\text{J}}} \gamma {\varvec{m}} \times {\varvec{\sigma}} + \alpha {\varvec{m}} \times \frac{{\partial {\varvec{m}}}}{\partial t}$$

11 $${\varvec{m}} \times \frac{{\partial {\varvec{m}}}}{\partial t} = - \gamma \mu_{0} {\varvec{m}} \times \left( {{\varvec{m}} \times {\varvec{H}}_{{{\mathbf{eff}}}} } \right) + a_{{\text{J}}} \gamma {\varvec{m}} \times {\varvec{\sigma}} - b_{{\text{J}}} \gamma {\varvec{m}} \times \left( {{\varvec{m}} \times {\varvec{\sigma}}} \right) - \alpha \frac{{\partial {\varvec{m}}}}{\partial t}$$

Combining Eq. (10) and Eq. (11):

12 $${\varvec{\tau}}_{{{\mathbf{SOT}}}} = \user2{ } - \frac{{\upgamma }}{{\left( {1 + {\upalpha }^{2} } \right)}}a_{{\text{J}}} \left[ {\left( {1 + \eta \alpha } \right){\varvec{m}} \times \left( {{\varvec{m}} \times {\varvec{\sigma}}} \right) + \left( {\eta - \alpha } \right)\left( {{\varvec{m}} \times {\varvec{\sigma}}} \right)} \right]$$

In MuMax3, the spin torques is expressed as:

13 $${\varvec{\tau}}_{{{\mathbf{SL}}}} = \gamma \frac{{\hbar J_{c} }}{{eM_{s} t_{F} }}\frac{{ \epsilon+ \alpha \epsilon^{\prime}}}{{\left( {1 + \alpha^{2} } \right)}}\left( {{\varvec{m}} \times \left( {{\varvec{m}}_{{\mathbf{P}}} \times {\varvec{m}}} \right)} \right) + \gamma \frac{{\hbar J_{c} }}{{eM_{s} t_{F} }}\frac{{\epsilon^{\prime} - \alpha \epsilon }}{{\left( {1 + \alpha^{2} } \right)}}\left( {{\varvec{m}}_{{\mathbf{P}}} \times {\varvec{m}}} \right)$$

14 $$\epsilon = \frac{{P\left( {{\text{r}},{\text{t}}} \right){\Lambda }^{2} }}{{\left( {{\Lambda }^{2} + 1} \right) + \left( {{\Lambda }^{2} - 1} \right)\left( {{\varvec{m}} \times {\varvec{\sigma}}} \right)}}$$

${\varvec{m}}_{{\mathbf{P}}}$ is the reference layer magnetization, *P* the spin polarization, $\epsilon^{\prime}$ the secondary Slonczewski STT term, ${\Lambda }$ the barrier layer thickness. We use Slonczewski spin transfer torque to replace spin orbit torque in Mumax3 by setting ${\Lambda }$ = 1, *P* = $\xi_{{{\text{DL}}}}$, $\epsilon {\prime } = \eta \epsilon$. And we take the exchange constant *A*_ex_ = 1 × 10^–11^ J/m, the PMA energy density *K*_u_ = 2.36 × 10^6^ J/m^3^.

### Supplementary Information

Supplementary Information.

## Data Availability

The authors declare that the data supporting the findings of this study are available within the article and are available from the corresponding author upon reasonable request.

## Code availability

The authors declare that all the codes used for the macro spin modeling presented in this study are available from the corresponding author upon reasonable request. The source code of MuMax3 is available at http://mumax.github.io/.

## References

[1] Shreya S, Jain A, Kaushik BKJMJ (2021). Computing-in-memory using voltage-controlled spin-orbit torque based MRAM array. Microelectron. J..

[2] Chiu Y.C (2023). A CMOS-integrated spintronic compute-in-memory macro for secure AI edge devices. Nat. Electron..

[3] Sebastian A, Le Gallo M, Khaddam-Aljameh R, Eleftheriou E (2020). Memory devices and applications for in-memory computing. Nat. Nanotechnol..

[4] Jung S (2022). A crossbar array of magnetoresistive memory devices for in-memory computing. Nature.

[5] Lanza M (2022). Memristive technologies for data storage, computation, encryption, and radio-frequency communication. Science.

[6] Devaraj, G. P. *et al.* Design and Analysis of Modified Pre-Charge Sensing Circuit for STT-MRAM. *2021 Third International Conference on Intelligent Communication Technologies and Virtual Mobile Networks (ICICV).IEEE.* 507–511 (2021).

[7] Bhatti S (2017). Spintronics based random access memory: A review. Mater. Today.

[8] Jain S, Ranjan A, Roy K, Raghunathan A (2018). Computing in memory with spin-transfer torque magnetic RAM. IEEE Trans. Very Large Scale Integr. Syst..

[9] Fong X (2016). Spin-transfer torque devices for logic and memory: Prospects and perspectives. IEEE Trans. Comput.-Aided Des. Integr. Circuits Syst..

[10] Kim C (2020). Spin-orbit torque driven magnetization switching and precession by manipulating thickness of CoFeB/W heterostructures. Adv. Electron. Mater..

[11] Gambardella P, Miron IM (2011). Current-induced spin–orbit torques. Philos. Trans. R. Soc..

[12] Zhang K (2020). Compact modeling and analysis of voltage-gated spin-orbit torque magnetic tunnel junction. IEEE Access.

[13] Lin H (2022). All-electrical control of compact SOT-MRAM: Toward highly efficient and reliable non-volatile in-memory computing. Micromachines.

[14] Shao Q (2021). Roadmap of spin-orbit torques. IEEE Trans. Magn..

[15] Ryu J, Lee S, Lee KJ, Park BG (2020). Current-induced spin-orbit torques for spintronic applications. Adv. Mater..

[16] Lopez-Dominguez V, Shao Y, Khalili AP (2023). Perspectives on field-free spin–orbit torque devices for memory and computing applications. J. Appl. Phys..

[17] Raymenants E (2021). All-electrical control of scaled spin logic devices based on domain wall motion. IEEE Trans. Electron Dev..

[18] Kumar R, Divyanshu D, Khan D, Amara S, Massoud Y (2022). Polymorphic hybrid CMOS-MTJ logic gates for hardware security applications. Electronics.

[19] Barla P, Joshi VK, Bhat S (2021). Design and analysis of SHE-assisted STT MTJ/CMOS logic gates. J. Comput. Electron..

[20] Huang X (2023). Implementing versatile programmable logic functions using two magnetization switching types in a single device. Adv. Funct. Mater..

[21] Zhang H, Kang W, Wang L, Wang KL, Zhao W (2017). Stateful reconfigurable logic via a single-voltage-gated spin hall-effect driven magnetic tunnel junction in a spintronic memory. IEEE Trans. Electron Dev..

[22] Wang J, Meng H, Wang J-P (2005). Programmable spintronics logic device based on a magnetic tunnel junction element. J. Appl. Phys..

[23] Rowlands GE (2019). A cryogenic spin-torque memory element with precessional magnetization dynamics. Sci. Rep..

[24] Grimaldi E (2020). Single-shot dynamics of spin–orbit torque and spin transfer torque switching in three-terminal magnetic tunnel junctions. Nat. Nanotechnol..

[25] Yamamoto T (2018). Thermally induced precession-orbit transition of magnetization in voltage-driven magnetization switching. Phys. Rev. Appl..

[26] Lee JM (2018). Oscillatory spin-orbit torque switching induced by field-like torques. Commun. Phys..

[27] Li X, Kang A, Liu Z, Zhou Y (2019). Ultrafast field-free magnetization switching using bi-directional spin Hall current and antiferromagnetic interlayer exchange. Appl. Phys. Lett..

[28] Kateel V (2023). Field-free spin-orbit torque driven switching of perpendicular magnetic tunnel junction through bending current. Nano Lett..

[29] Xue F (2023). Field-free spin-orbit torque switching assisted by in-plane unconventional spin torque in ultrathin [Pt/Co]N. Nat. Commun..

[30] Zheng Z (2021). Field-free spin-orbit torque-induced switching of perpendicular magnetization in a ferrimagnetic layer with a vertical composition gradient. Nat. Commun..

[31] Legrand W, Ramaswamy R, Mishra R, Yang H (2015). Coherent subnanosecond switching of perpendicular magnetization by the fieldlike spin-orbit torque without an external magnetic field. Phys. Rev. Appl..

[32] Zhu D, Zhao W (2020). Threshold current density for perpendicular magnetization switching through spin-orbit torque. Phys. Rev. Appl..

[33] Lakshmanan M (2011). The fascinating world of the Landau–Lifshitz–Gilbert equation: An overview. Philos. Trans. R. Soc..

[34] Koo HC (2020). Rashba effect in functional spintronic devices. Adv. Mater..

[35] Decker MM (2017). Time resolved measurements of the switching trajectory of Pt/Coelements induced by spin-orbit torques. Phys. Rev. Lett..

[36] Baumgartner M (2017). Spatially and time-resolved magnetization dynamics driven by spin–orbit torques. Nat. Nanotechnol..

[37] Yoon J (2017). Anomalous spin-orbit torque switching due to field-like torque–assisted domain wall reflection. Sci. Adv..

[38] Fan W (2019). Asymmetric spin-orbit-torque-induced magnetization switching with a noncollinear in-plane assisting magnetic field. Phys. Rev. Appl..

[39] Vansteenkiste A (2014). The design and verification of MuMax3. AIP Adv..

[40] Wan C (2017). Programmable spin logic based on spin hall effect in a single device. Adv. Electron. Mater..

[41] Park J, Rowlands GE, Lee OJ, Ralph DC, Buhrman RA (2014). Macrospin modeling of sub-ns pulse switching of perpendicularly magnetized free layer via spin-orbit torques for cryogenic memory applications. Appl. Phys. Lett..

