# Supplementary material for: Reconfigurable spintronic logic gate utilizing precessional magnetization switching
Source: Sci Rep. 2024 Jun 26;14:14796. doi: 10.1038/s41598-024-65634-9 (PMC11208557; doi:10.1038/s41598-024-65634-9)
Supplement: Supplementary file 1 — Supplementary Information. [file 41598_2024_65634_MOESM1_ESM.pdf]

Supplementary Material

Reconfigurable spintronic logic gate utilizing precessional magnetization switching

Ting Liu[1], Xiaoguang Li[1,*], Hongyu An[2], Shi Chen[1], Yuelei Zhao[3], Sheng Yang[3], Xiaohong Xu[4,5], Cangtao Zhou[1], Hua Zhang[1,*], Yan Zhou[3,*]

[1] *College of Engineering Physics, and Shenzhen Key Laboratory of Ultraintense Laser and Advanced Material Technology, Shenzhen Technology University, Shenzhen, 518118, China*

[2] *College of New Materials and New Energies, Shenzhen Technology University, Shenzhen, 518118, China*

[3]*School of Science and Engineering, The Chinese University of Hong Kong, Shenzhen, 518172, China.*

[4]*Research Institute of Materials Science of Shanxi Normal University & Collaborative Innovation Center for Shanxi Advanced Permanent Magnetic Materials and Technology, Linfen, 041004, China.*

[5]*School of Chemistry and Materials Science of Shanxi Normal University & Key Laboratory of Magnetic Molecules and Magnetic Information Materials of Ministry of Education, Linfen, 041004, China.*

Corresponding Authors：

* E-mail: lixiaoguang@sztu.edu.cn

* E-mail: zhanghua@ sztu.edu.cn

* E-mail: zhouyan@cuhk.edu.cn

Switching stability

We added thermal fluctuation at 5ns before the current is applied, and the precessional switching characteristic is not qualitatively affected, as shown in Fig. S1. We note that the temperature (300K) gives rise to small variations of initial orientation of the magnetization, and slightly reduces the threshold current density. However, the critical current variability, as shown in Fig. S1, ensures the feasibility of implementing logic gate under thermal fluctuations. Moreover, at zero temperature, the threshold current density is about 0.1×10^{12} A/m^2 lower than that at room temperature when pulse width is 0.7 ns. As the pulse width decreases, the difference between the two threshold currents increases.

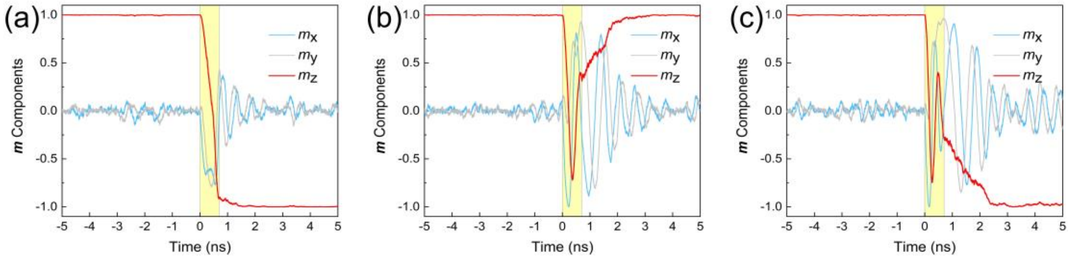

Figure S1. Precessional magnetization switching processes under room temperature (300K). The thermal fluctuation is introduced at t = -5 ns, and then current is applied at t = 0 ns. The duration of the current pulse is highlighted by the yellow part. The current pulse width T_c = 0.7 ns, and the current density J_c = (a) 0.94×10^{12} A/m^2, (b) 1.24×10^{12} A/m^2 and (c) 1.7×10^{12} A/m^2.

The nonpolar switching

Because no external field is introduced to break the switching symmetry, the oscillatory switching of the free layer is nonpolar. The simulation results below also indicate that the magnetization switching is symmetric. Fig. S2 (a), (b), (c) show the switching process with the initial magnetization being (0, 0, 1), while Fig. S2 (d), (e), (f) show that with the initial magnetization being (0, 0, -1).

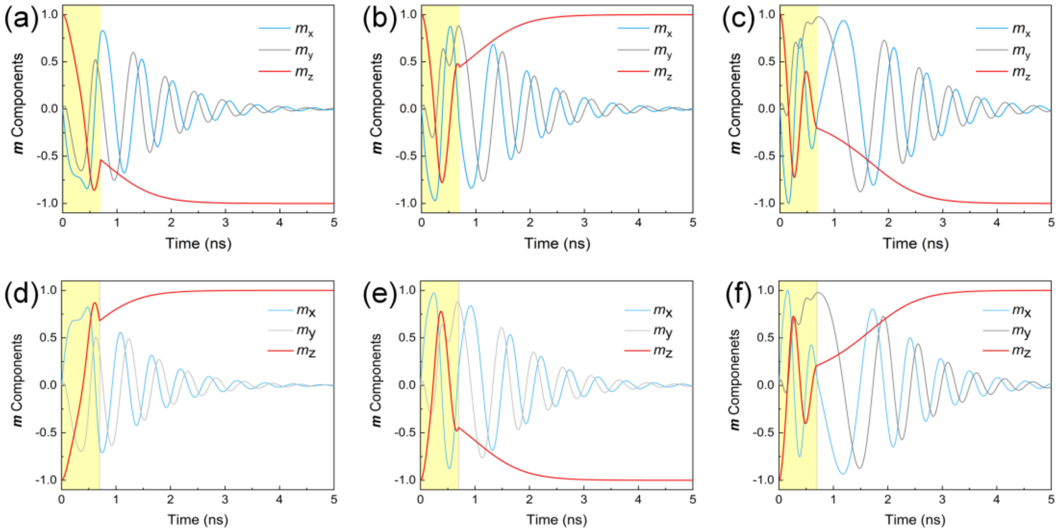

Figure S2. Precessional magnetization switching processes driven by the combined damping-like and field-like SOT. (a)-(c) Evolution of the magnetization components with time while the initial state being (0, 0, 1). The duration of the current pulse is highlighted by the yellow part. The current pulse width T_c = 0.7 ns, and the current density J_c = (a) 0.94×10^{12} A/m^2, (b) 1.24×10^{12} A/m^2 and (c) 1.7×10^{12} A/m^2. (d)-(f) The initial magnetization state being (0, 0, -1) corresponding to (a)-(c).
